# Cervicofacial Necrotizing Fasciitis Originating From Odontogenic Infections: A Report of Two Cases

**DOI:** 10.7759/cureus.70305

**Published:** 2024-09-27

**Authors:** Hanan Wahbi, Raqi Barber, Sana Mando, Raghed Eid, Nabil Kochaji

**Affiliations:** 1 Oral Pathology and Histology, Damascus University, Damascus, SYR; 2 Dentistry, Damascus University, Damascus, SYR; 3 Oral Maxillofacial Surgery, Tishreen University, Lattakia, SYR

**Keywords:** cervicofacial region, cnf, necrosis, necrotizing fasciitis, odontogenic infection

## Abstract

Cervicofacial necrotizing fasciitis (CNF) is infrequently reported to originate from odontogenic infections. Even with such rarity, its development is potentially life-threatening. The current report aims to demonstrate how severe and risky the consequences of odontogenic infections are. This report presents two clinically diagnosed necrotizing fasciitis (NF) cases. The first case is a 33-year-old man suffering diffusing pain upon palpating submandibular regions with necrotic black skin after experiencing red skin and gaseous infiltration. He required skin grafting, post-healing esthetic surgeries. The second case is a 14-year-old girl with malnutrition who displayed evident necrosis in the soft tissue of the cervicofacial region. Bacteriological examinations were done, and intravenous antibiotic treatment was administered to the patient. This case highlights that dental infections are probably found to activate serious complications in certain cases; early clinical diagnosis is extremely essential in such cases. Besides, immediate surgical interference, accompanied by antibiotic therapy, plays a decisive role in treatment success.

## Introduction

Necrotizing soft-tissue infections (NSTIs) contain fasciitis, myositis, and cellulitis. Diagnosing necrotizing fasciitis (NF) infections is not easy. In the early stages, cases of NF are easily confused with cellulitis or abscesses. Although rare and highly progressive, (NF) can spread rapidly resulting in skin destruction and necrosis to subcutaneous fat and fascia [1]. The term “necrotizing fasciitis” was initially introduced in 1952, when Wilson intended to describe prevalent necrosis attacking the layer of superficial fascia. Later in the literature, this term was widely accepted. In fact, (NF) affects individuals regardless of their age or gender [2].

CT and MRI may show edema extending along the fascial plane. Although these findings may have been absent in the early stages of (NF), it is found in cellulitis and abscess, along with gaseous infiltration of subcutaneous tissue sometime in the early stages. Although (MRI) may provide superior results, (CT) is favored as the initial imaging choice because, in general, it is generally more readily available for emergent imaging than (MRI). Another benefit is that (CT) can be possessed promptly [1-2].

However, one of the regional manifestations of this necrotic disease, known as cervicofacial necrotizing fasciitis (CNF), has rarely been reported; it accounts for only 10% of all diagnosed cases. Nonetheless, this severe type leads to substantial levels of morbidity and high rates of mortality (about 40%). This disease is known to occur in both healthy and chronically ill patients following minor trauma or surgery [3]. Being rare, (CNF) typically results from a host of factors, including dental infection, infections contingent on trauma, throat abscess, and osteoradionecrosis (ORN).

This disease is associated with other health complications as well. In certain, but rare, cases, it can be caused by infections of salivary glands. Due to high vascularity of the head and neck region, (CNF) accounts for approximately 3-4% of all body cases [4]. Zamir et al. concluded that diabetes mellitus is the most common immune-compromised comorbidity presented in a study of infections from dental origion extending in fascial spaces [5].

On the other hand, development of cervical necrotizing fasciitis is perceived as life-threatening, associated with mortali­ty rates of 7-20%. A strong link exists between these two percentages and the extent during which the infection is diagnosed [6]. Therefore, this report emphasizes the im­portance of immediate diagnosis, and aims at showing the serious consequences activated by dental infection. Other associative diseases, such as cellulitis and abscess of dental origin, should not be underestimated [7].

In its initial stages, (NF) was misdiagnosed in approximately three quarters of the reported patients. There exist predefined indicators endorsing early diagnosis, establishing a statistically reliable link between prompt intervention and reduction in mortality levels and limb-amputation rates. (NF)-diagnosed patients usually suffer from a clinically noticeable triad of pain, swelling and erythema, which are generally misdiagnosed as cellulitis or abscess. In its early manifestation, (NF) features deep pain, gradually exhibiting swelling and erythema [8,9].

NF classifications

NF is an infection attacking soft tissues, which leads to progressive destruction of the muscle’s fascia, along with the subcutaneous fat. Two major (NF) classifications are generally recognized in medicine circles, yet new classifications have recently been suggested to yield additional sub-types. However, the following two classifications are based on the underlying bacteria that initiate injury cascades [10].

Polymicrobial (Type I) NF: Type I NF originates from infections categorized as polymicrobial; they are identified via microbiological culture. This type of infection usually stems from aerobic and anaerobic bacteria. Complex microbiological profiles of attacking organisms lead to gaseous infiltration of subcutaneous tissue, which is closely analogous to gas gangrene. Most of the reported cases admit Type I NF; this type is prominently prevalent in older patients who suffer from chronic diseases [11].

Monomicrobial (Type II) NF: Type II NF is commonly associated with gram-positive organisms, such as group-A streptococcus (GAS) and methicillin-resistant Staphylococcus aureus (MRSA). Endotoxins released via Type-II-NF organisms are associated with clinical indications, like toxic shock syndrome. However, this type is not found to be associated with specific age groups. Many patients do not show comorbidities or portals of entry to make them susceptible to severe infections [11-13].

## Case presentation

Case 1

**Figure 1:**
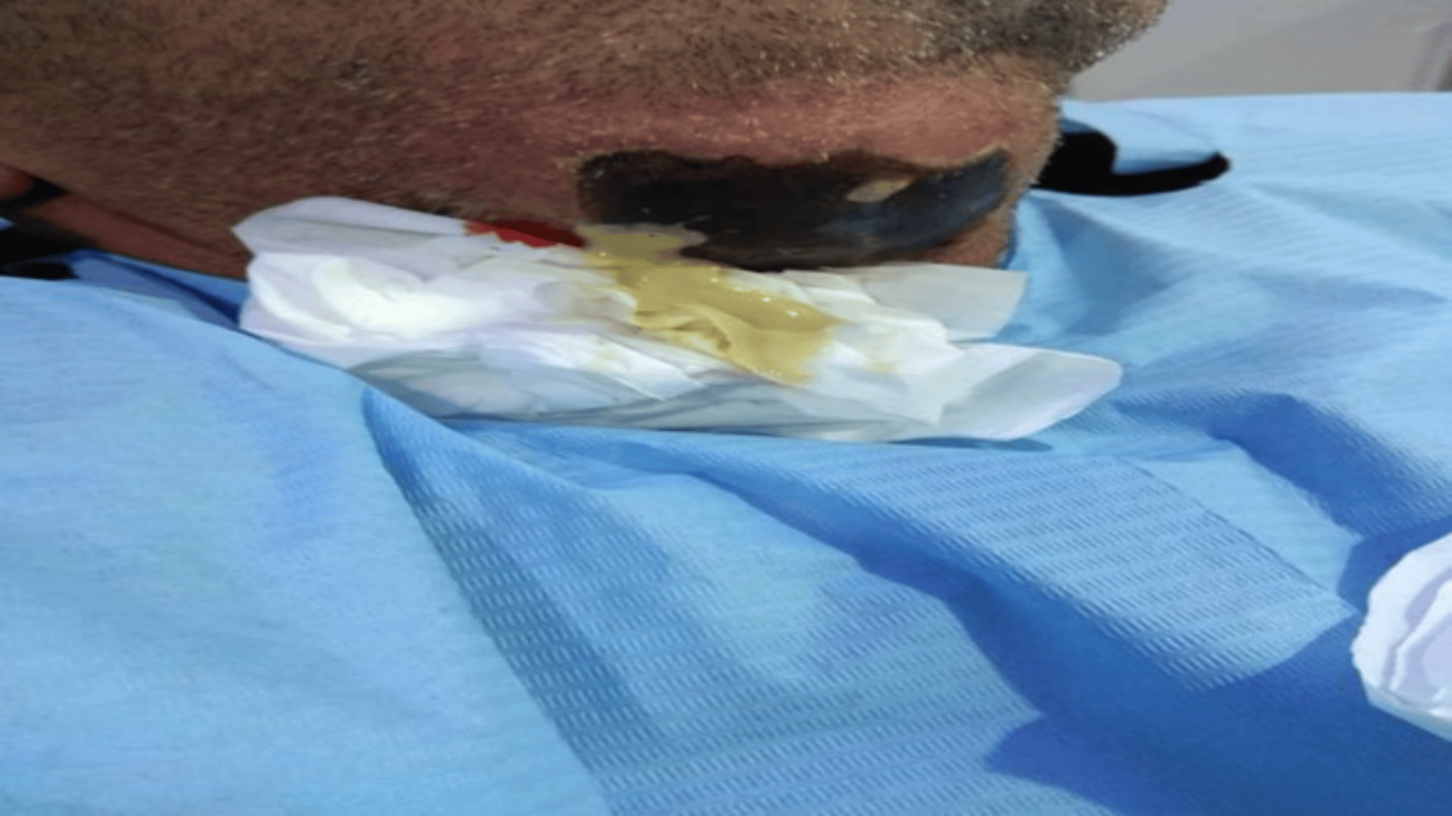
Black necrotic skin after 24 hours of swelling

The first case is a 33-year-old man suffering from malnutrition who consulted the researcher’s clinic for tooth pain and swelling in both submandibular and sublingual regions. He also had a fever. The only symptom the patient had five days prior to the consultation was pain in teeth 46 and 47. The initial examination revealed ma­croscopic tooth decay, lockjaw or trismus, mouth floor inflammation and widespread pain upon palpating the sub­mandibular region. He also displayed necrotic black skin, after red skin, and gaseous infiltration as illustrated in Figure 1.

**Figure 2:**
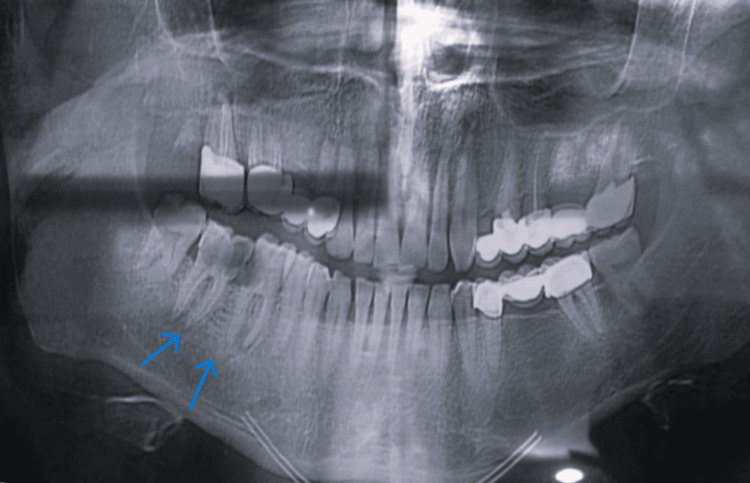
Panoramic radiograph The image shows decayed teeth 46 and 47 and abscess lesions (blue arrows).

At the time of hospital admission, the patient's temperature was 39°C and blood pressure was 120/75 mmHg. Examination of the heart revealed no abnormalities. No removed tissue was tested at the pathological laboratory, and only a small amount of pus was sent for testing. Following the conduction of a bacteriological examination, intravenous antibiotic treatment was administered along with panoramic imaging of the area. Figure 2 displays a panoramic image, proving the presence of apical lesions on teeth (67-46). 

**Figure 3:**
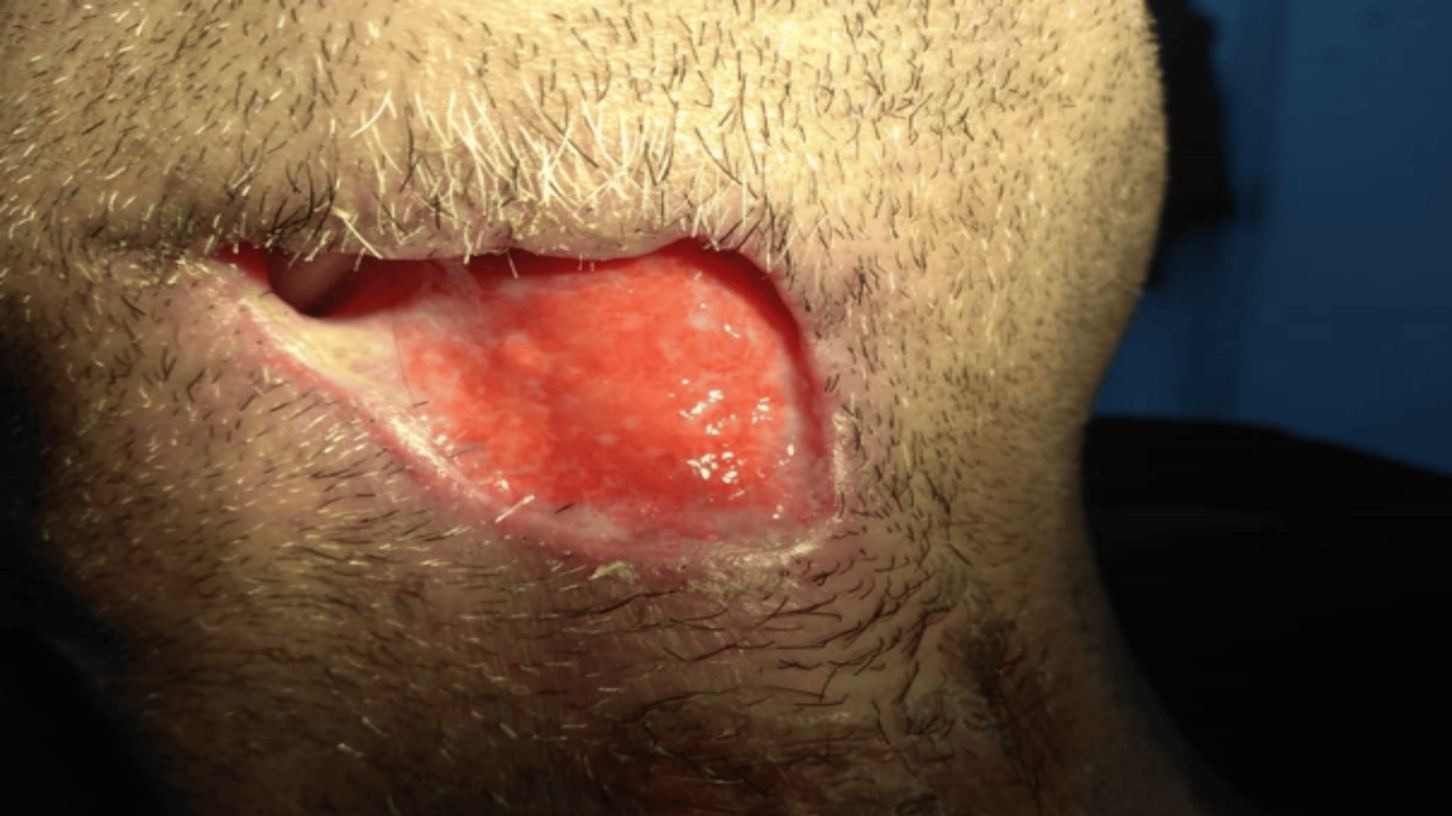
Mandibular edge after removing necrotic tissues

A few hours later, maxillofacial surgery was performed to treat surgical drainage of the abscess, as illustrated in Figure 3. After resection of the underlying necrotic tissue and removal of the necrotic skin, long treatment with intravenous broad-spectrum antibiotics was administered: a high dose of penicillin 1000 mg twice a day (BID), a high dose of clindamycin 300 mg BID, metronidazole 500 mg thrice a day (TID), and paracetamol 1000 mg were administered for the three-day stay in the hospital. In addition, clindamycin 300 mg BID and augmentin 1000 mg BID were prescribed for 12 days after discharge.

**Figure 4:**
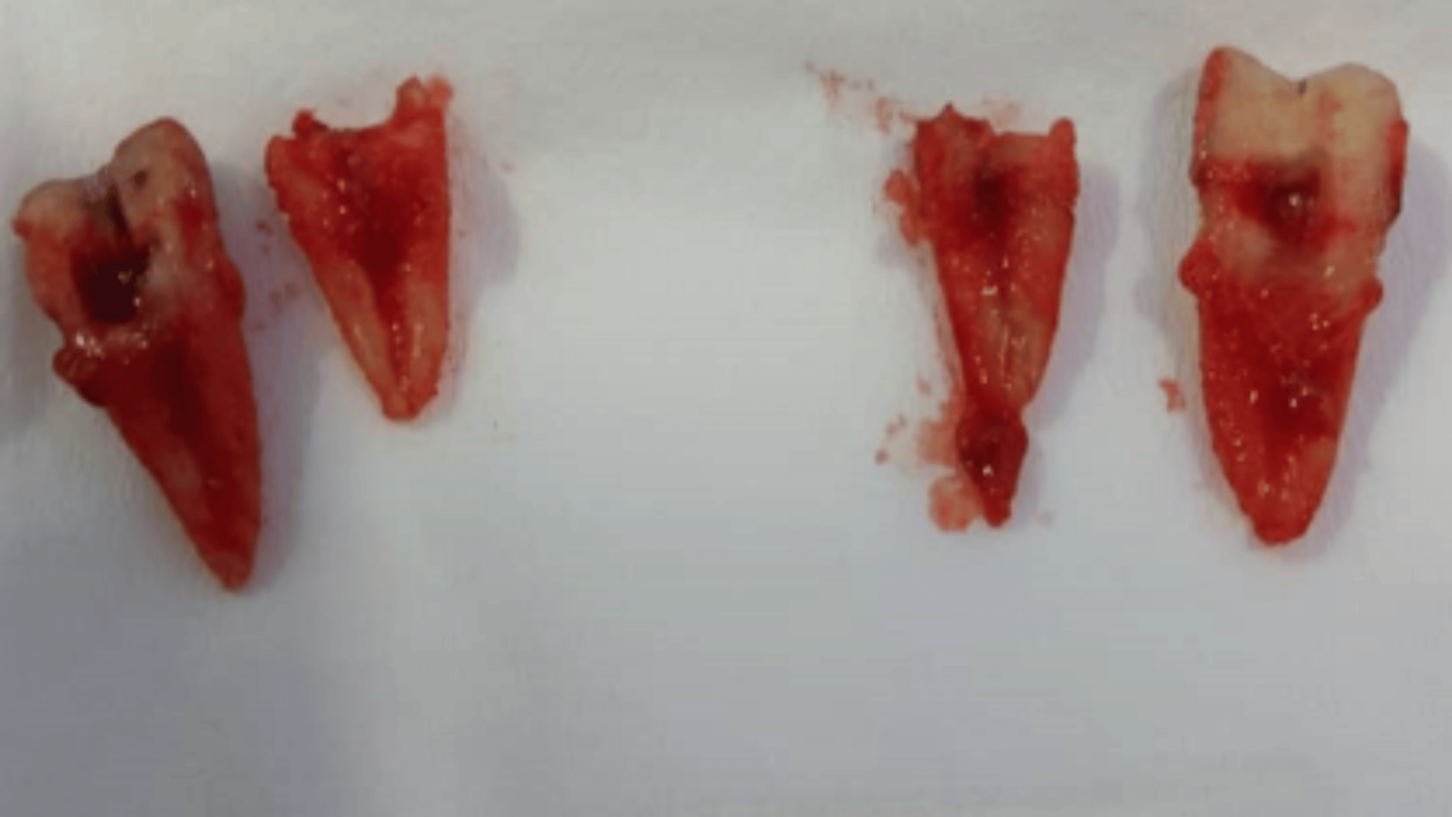
Extraction of teeth 46 and 47

**Figure 5:**
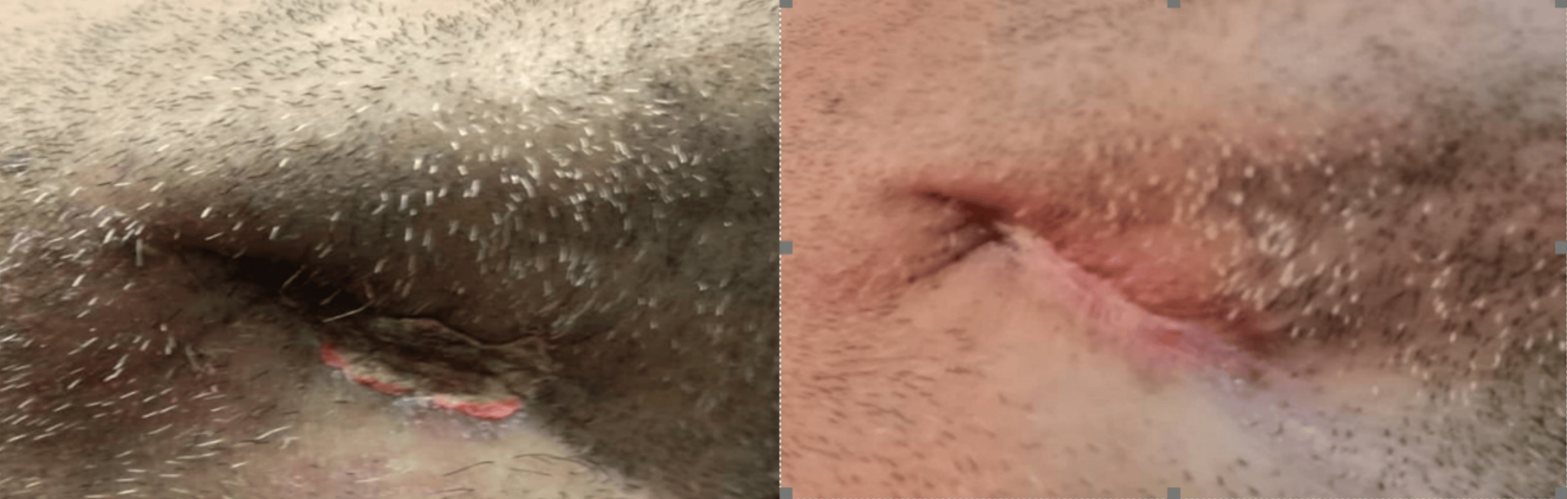
Post-healing large scar images Right-hand side image: after one month; Left-hand side image: after three months.

In this study, it was noticed that streptococcus strains were the most frequent species in bacterial culture. No adjustment was made to the medications administered after the bacterial transplant; only the medical treatment plan was applied. Three days after the surgical debridement, extraction was performed under local anesthesia at the areas of the first and second lower right molars Figure 4. Accordingly, improvement was noticed in the post-surgery stage. In one month's time, the patient showed signs of improvement; however, a bad post-healing scar was visible, Figure 5.

This study recommends that skin grafting or esthetic surgeries be performed in this manner due to the existence of a muscle breakdown. The patient's scar was repaired by way of plastic surgery. It was not possible to obtain the photo of the repaired scar for reasons beyond control.

Case 2

The second case is a 14-year-old girl suffering from malnutrition. She consulted the emergency department of the hospital upon feeling excruciating tooth pain and swelling in the left submandibular region. Upon admission, her temperature was 38.8°C, and her blood pressure was 120/75 mmHg. The patient has reported that her symptoms started to appear two days prior to her admission. After clinical examination, the case was diagnosed with trismus and hardening of the mouth floor tissues.

**Figure 6:**
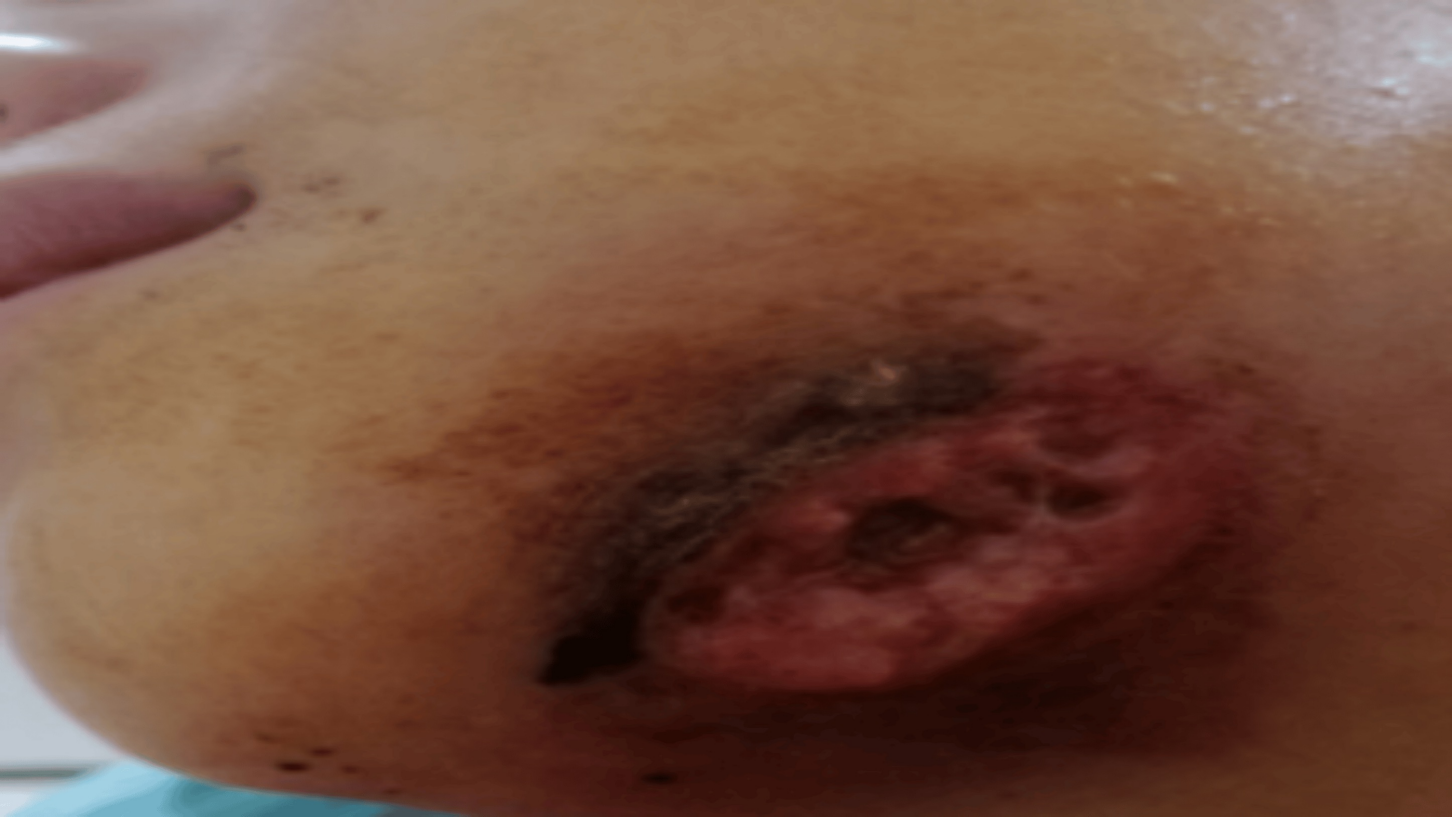
Black necrotic skin

The physical examination showed that the swelling extended across the cervical region, with evident necrotic soft-tissue areas and subcutaneous emphysema. The panoramic images showed severe infection, which extended from tooth 36 to the posterior-anterior facial axis. To address the case clinically, the patient was admitted to the intensive care unit, and an immediate bacteriological examination was carried out. Maxillofacial surgery was performed on the same night in order to treat the drainage of the abscess. At a later stage, resection of the underlying necrotic tissue, removal of necrotic skin, and cleansing of the infected area were performed (Figure 6).

Over a three-day stay in the hospital, treatment of intravenous broad-spectrum antibio­tics was administered with a high dose of (metronidazole 500 mg TID intravenously (IV), augmentin 1000 mg BID IV, and clindamycin 300 mg BID, augmentin 1000 mg .cap., bid. Profen 400 mg bid) were administered. As for the pus culture, the results showed a single bacterial strain (hemolytic streptococcal infection).

**Figure 7:**
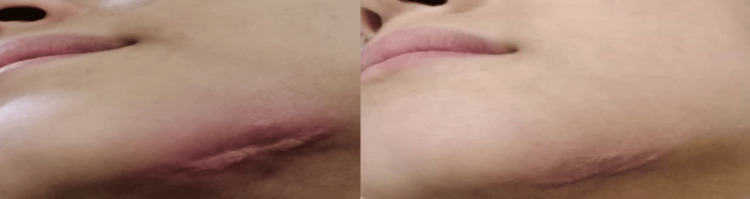
Post-treatment images A small scar was left after one month of treatment (left-hand side image) and after two months (right-hand side image).

The patient showed favorable healing progress the following day and was discharged after three days of stay. Recovery was attained after one month, sustaining a small scar (Figure 7). This report suggests that skin grafting or esthetic surgeries are not recommended due to the fact that no muscle destruction was noticed. The patient was advised to undertake plastic surgery; however, she did not go along with the idea.

While previous studies concluded that multiple bacterial strains were involved in the development of NF, this report maintains that a single bacterial strain was identified as the most responsible bacterial factor for the development of NF. There are two possible explanations for these different results. The antibiotic treatment administered prior to biological sample collection (first spiramycin 1500 IU; second amoxicillin 500 mg, along with other antibiotics) might have eliminated the antibiotic-sensitive bacterial flora, which allowed for the excessive development of a single bacterial strain.

## Discussion

The main aim of this two-case report is to emphasize the serious consequences provoked by dental infection. Early diagnosis, prompt surgical drainage and appropriate medical treatment characterize a key means to increasing survival rates [13]. However, 2.6% of all dental infections develop NF, and several reports confirmed that the immense spread of the infection and wound complications could commonly result if risk factors are present. These risk factors include diabetes, malignancy, steroid use, vascular deficiency, HIV, alcoholism, obesity or even bad nutrition [14].

NF provokes large scars that necessitate long-term treatment, sometimes represented in skin grafting or esthetic surgeries, required in the post-healing phase [15]. Analysis of odontogenic infections conducted by other researchers indicates the threshold of odontogenic necrotizing fasciitis to start in mandibular molar sites. A similar result was found in this current two-case report, which reiterates that mandibular molars are the major threshold of necrotizing fasciitis. The fact that the submandibular region is in close proximity with mandibular molars can explain that it was first affected by the septic process, from whose level, the infection extended to the facial level. Hence, early effective treatment of odontogenic submandibular infections is imperative in the prevention and treatment of necrotizing fasciitis [16,17].

## Conclusions

Surgical treatment and antibiotic therapy play a decisive role in the status of odontogenic necrotizing fasciitis. Dental infection could trigger serious complications in a number of reported cases. Early diagnosis, prompt surgical drainage, and appropriate medical treatment characterize a key means to increase patients’ overall survival rates.
